# The Underlying Mechanism of Poisoning after the Accidental Inhalation of Aerosolised Waterproofing Spray

**DOI:** 10.3390/jox14020039

**Published:** 2024-05-28

**Authors:** Alexander C. Ø. Jensen, Niels E. Ebbehøj, Anja J. Huusom, Keld A. Jensen, Ulla B. Vogel, Jorid B. Sørli

**Affiliations:** 1 The National Research Centre for the Working Environment, 2100 Copenhagen, Denmarkkaj@nfa.dk (K.A.J.); ubv@nfa.dk (U.B.V.); 2 Department of Occupational Health and Social Medicine, Holbæk Hospital, 4300 Holbæk, Denmark; ebbehoj@dadlnet.dk; 3 Department of Occupational and Environmental Medicine, Copenhagen University Hospital, Bispebjerg and Frederiksberg, 2400 Copenhagen, Denmark; anja.julie.huusom@regionh.dk

**Keywords:** pulmonary surfactant, acute airway toxicity, impregnation spray

## Abstract

Waterproofing sprays can cause acute respiratory symptoms after inhalation, including coughing and dyspnoea shortly after use. Here, we describe two cases where persons used the same brand of waterproofing spray product. In both cases the persons followed the instructions on the product and maximized the ventilation by opening windows and doors; however, they still became affected during the application of the product. Products with the same batch number as that used in one case were tested for their effect on respiration patterns of mice in whole-body plethysmographs and lung surfactant function inhibition in vitro. The product was used in spraying experiments to determine the particle size distribution of the aerosol, both using a can from one case and a can with an identical batch number. In addition, the aerosols in the mouse exposure chamber were measured. Aerosol data from a small-scale exposure chamber and data on the physical and temporal dimensions of the spraying during one case were used to estimate the deposited dose during the spraying events. All collected data point to the spraying of the waterproofing product being the reason that two people became ill, and that the inhibition of lung surfactant function was a key component of this illness.

## 1. Introduction

Waterproofing spray products frequently cause harm to persons using them, and case studies describing events where one to hundreds of people have been exposed to them and become ill can be found in literature (e.g., [1,2,3,4,5]). Usually, the person using the product has followed the instructions provided by the supplier to the best of their ability, e.g., when used at home, the person has opened windows and doors to maximize ventilation. Despite following instructions, some users experience adverse health effects after using the products. Waterproofing products are produced for the surface treatment of different materials, such as textiles, furniture, outdoor equipment, or building materials. They are popular as they introduce many desirable qualities, such as increasing the lifetime of the treated surface, make cleaning easier, and making surfaces water-repellent. Waterproofing products are often formulated as sprays, as this eases application on large or uneven surfaces [6]. However, spraying also creates aerosols, in the form of tiny droplets, in the air during and after application as a direct result of spraying and overspray. Depending on the formulation, particles may also change size during and after application due to condensation and reaction processes [7,8]. These droplets can stay suspended in the air for an extended period of time after spraying and be inhaled and deposit in the lungs either during or after spraying. The product can potentially have adverse effects on the lungs. Symptoms often appear during or shortly after spray application. The symptoms resolve after a few days, but sometimes have long lasting effects on the lungs, usually when there is an underlying pathology of the lungs (e.g., [3,9,10]). 

We have previously suggested that the biological target of poisonings by the inhalation of waterproofing products is the lung surfactant in the respiratory parts of the lungs [11]. Lung surfactant is secreted into the thin liquid lining covering the respiratory parts of the lungs, where it regulates surface tension at the air–liquid interface in the alveoli [12,13]. The regulation of surface tension during breathing is essential for normal lung function, and the disruption of lung surfactant function can lead to decreased lung function, as described in an adverse outcome pathway [14] and https://aopwiki.org/aops/302 (accessed on 1 March 2024). 

In this paper, we describe two independent cases where persons became ill after using the product Guardian textile protection spray. We measured the particle size distribution and concentration of the aerosols generated while using the product, and the effect of the product on the respiration of exposed mice. Finally, we tested the effect of Guardian textile protection on lung surfactant function in vitro. 

## 2. Materials and Methods

### 2.1. Human Exposure Cases

The two persons exposed to Guardian textile protection spray during use were referred to the Danish Poisons Information Centre (DPIC) at Bispebjerg hospital by the medical professionals they contacted. The DPIC collected description of the events and medical histories. In both cases, the product used was identified as Guardian textile protection spray; in one case (A) the original product was sent to the DPIC, and cans of the product with identical production IDs were acquired. 

### 2.2. Chemical Composition of the Product

The product chemical composition changed in 2020; the tests described here were performed on the formulation used before 2020. According to the product information sheet, the tested formulation of Guardian textile protection contained 80–95 w/w% hydrated heavy naphtha (CAS 64742-48-9), 5–10 w/w% carbon dioxide (CAS 124-38-9), and 0–1 w/w% dipropylene glycol monomethyl ether (CAS 34590-94-8).

### 2.3. Animals

The mouse strain BALB/cJ used in this study was chosen because previous inhalation experiments have been performed on this strain, and this allows comparisons to be made with previous data. Therefore, 34 inbred male BALB/cJ mice (Taconic M&B, Ry, Denmark) were housed in polypropylene cages (1290D Eurostandard type III from Scanbur, Karslunde, Denmark, 425 mm × 266 mm × 155 mm) furnished with aspen bedding material, and enriched with small aspen blocks (both from Tapvei, Paekna, Estonia). The mice were 4–5 weeks old when they arrived, and they were subsequently acclimatised for a minimum of one week. The light period was from 06:00 to 18:00, and the experiments were performed between 10:00 and 15:00. The temperature during housing was 21.5 ± 0.8 °C and the relative humidity was 40.3 ± 4.6% (mean ±standard deviation (sd)). Cages were sanitised twice weekly. Food (Altromin no. 1324, Altromin, Lage, Germany) and tap water were available ad libitum. The mice were randomly assigned to cages upon arrival, with 3–6 mice per cage. 

### 2.4. Ethical Statement

All experiments were performed by trained personnel and conformed to the Danish Regulations on Animal Experiments [15,16], which include guidelines for the care and use of animals in research, and according to EU Directive 2010/63 [17]. Anaesthesia was not used during the experiments, because the measurement of breathing patterns depends on the animals being fully awake with uncompromised breathing. If an animal had a sudden and persistent drop in tidal volume, it was removed from the exposure and killed with cervical dislocation. All other animals were killed immediately after end of exposure by cervical dislocation. We did not examine the lungs for histological changes at a later time point, as earlier experiments have showed that the exposure to waterproofing sprays can be fatal, and this is not permitted for animal ethical reasons. 

### 2.5. In Vivo Exposure of Guardian Textile Protection

The mice were weighed and placed in plethysmographs of their corresponding size. The plethysmographs allow for nose-only exposure, as only the head is placed in the chamber. The plethysmographs were placed with the mice head positioned in a 20 L stainless steel and glass exposure chamber with an air flow of 20 L/min, and an air exchange rate of approximately one per min. Their breathing was monitored throughout the experiment in real-time using Notocord-hem data acquisition software (version 4.4.0.1, Instem, Staffordshire, UK). The mice were given clean air to breathe for 15 min prior to initiation of exposure. As the respiratory parameters change during the first 5 min [18], we used the last 10 min prior to the start of exposure as the baseline measure. Animals that did not have a stable baseline measure were removed prior to starting the exposure. Animals that attempted escape by pulling the head into the plethysmograph were adjusted and continued in the experiment. 

After the baseline period, the mice were exposed to an aerosol of Guardian textile protection. Immediately prior to the experiment, the product was sprayed into a glass beaker, and the liquid drawn into a glass syringe. The syringe was placed in an infusion pump (Legato 100, Buch & Holm A/S, Denmark) and connected via plastic tubing to a Pitt no. 1 jet nebulizer [19], where aerosols were generated by pressurised air. The aerosol concentration was changed by adjusting the infusion rate into the nebuliser. Groups of mice were exposed to Guardian textile protection at an infusion rate of 0.1 mL/min (60 min), 0.4 mL/min (60 min), or 0.6 mL/min (15 min). The control group was exposed to 0.9% NaCl in water for 60 min at different infusion rates (0.025 mL/min (15 min), 0.05 mL/min (15 min), 0.1 mL/min (15 min), and 0.5 (15 min)). After the end of the exposures, the data were analysed for changes in breathing parameters. Changes in several breathing parameters were analysed, and a comprehensive description of breathing pattern analysis and interpretation has been reported elsewhere [20,21]. 

### 2.6. In Vitro Exposure of Lung Surfactant in the Constrained Drop Surfactometer

Guardian textile protection was aerosolised as described for in vivo exposure, but led into the 1.9 L exposure chamber of the constrained drop surfactometer (CDS, BioSurface Instruments, Honolulu, HI, USA) via glass tubing. It was tested at an infusion rate of 0.4 mL/min, the lowest infusion rate that causes a change in tidal volume (VT) in exposed mice. The lung surfactant, Curosurf (Chiesi, Parma, Italy), was adjusted to a concentration of 2.5 mg/mL in a buffer containing 0.9% NaCl, 1.5 mM CaCl_2_, and 2.5 mM HEPES, adjusted to pH 7.0. A drop of 10 µL lung surfactant was placed on a hollow pedestal with a diameter of 4 mm, and buffer was added and removed from the drop using a computerised syringe connected to the hollow pedestal at 3 sec cycles [22]. During the experiment, a camera took ten pictures per sec of the backlit drop. ADSA (axisymmetric drop shape analysis) software (version 4.0) [23] was used to analyse the pictures to calculate the surface tension of the droplet. After 20 sec of baseline, where the drop was exposed to clean air, the infusion of Guardian textile protection was started at 0.4 mL/min. The bottom of the chamber had hollow channels where the aerosols exited the chamber via holes. This air was passed through a HEPA filter before leading it into a fume hood. The drop was exposed for 3 min, and the experiment was repeated 5 times. The exposure was monitored using a quartz crystal microbalance (QCM, Vitrocell, Waldkirch, Germany) placed close to the drop. The drop was compressed by 12.4 ± 3.3% (mean ± sd). Lung surfactant function was defined as being inhibited if at least three consecutive minima in surface tension were larger than 10 mN/m. The time of inhibition, i.e., the first minimum above 10 mN/m, was combined with data from the QCM to estimate the inhibitory dose. The mass measured by the QCM was converted to the mass deposited on the lung surfactant droplet by multiplying with the average surface area of the drop throughout the experiments (0.18 cm^2^).

### 2.7. Aerosol Measurements

Aerosol number concentrations and aerodynamic particle size distributions from 6 nm to 10 μm were measured using an Electrical Low-Pressure Impactor (ELPI, Dekati, Finland) in 14 channels with a 1 sec time resolution. 

Measurements of the generated aerosol of Guardian textile protection were conducted during three different experiments. Experiment 1 was conducted in a standard fume hood to determine the direct emission from the spray can; the spray jet was directed to the inlet of the aerosol instrument (ELPI) such that the inlet was positioned at the centre of the spray jet to measure the particle size distribution from the middle of the spray cone. Two different spray cans were used to establish particle size distributions, the original spray can that was recovered from case A, and a spray can with the same batch number. The can was placed approximately 20 cm in front of the ELPI inlet, and the nozzle depressed for 25 s; this was done 3 times for the spray can from case A and 5 times for the spray can with the same batch number. 

Experiment 2 was conducted in the in vivo exposure chamber to determine the particle size distribution that the mice were exposed to during the in vivo exposure and followed the same procedure as described for these experiments, i.e., these aerosols were not from the spray can, but instead generated by the pressurised air nebuliser, as described above. The ELPI inlet was placed in a similar position to where the nose of the mouse would be in the chamber during exposure. 

Experiment 3 was conducted in a small-scale exposure chamber of 0.55 m^3^ with controlled ventilation to determine average generation rates for the modelling of the spray events and the calculation of the lung-deposited doses. The experimental set-up, the method for calculating the size-resolved emission rate, and the parameters for the modelling of exposure follow what has been described in detail in [11].

### 2.8. Statistics

To compare the results from the different exposed groups of mice, the tidal volume (VT) and time of break (TB) after 15 min exposure to 0.6 mL/min Guardian textile protection were compared to the same time point of 0.9% NaCl exposure. Similarly, the VT and TB after 60 min exposure in the 0.4 mL/min and 0.1 mL/min exposed groups were compared to the 0.9% NaCl exposed group. The statistical test was performed using RStudio version 2022.07.2. The groups were compared using the Mann–Whitney–Wilcoxon test as the data were not normally distributed. 

## 3. Results

### 3.1. Description of Poisoning Cases

#### 3.1.1. Case A

A person had bought a new sofa, and to protect it from stains, they sprayed it before use. They purchased three cans of Guardian textile protection spray and used two and most of the third can to treat the new sofa. They sprayed the sofa for approximately 30 min, and the windows in the living room were open throughout the spraying. After spraying, they felt pins-and-needles-like pain in their tongue and throat, combined with a headache and alternating between sweating and freezing. By the next day, the person had developed heart palpitations and diarrhoea. Four days after the exposure, the person was admitted to hospital with pain during breathing and a burning sensation in the lungs. They did not have any cough or flu symptoms, and the oxygen saturation, arterial carbon monoxide, and lactate values were all in the normal range. Electrocardiography and an X-ray of the lungs did not show any abnormal findings. The respiratory frequency was within the normal range (14–15 breaths per minute), and the person was sent home without treatment. The DPIC received what remained in the third can. Additional cans with the same production number (PRD 10 091117) were subsequently used for testing the product. 

#### 3.1.2. Case B

A person bought a new sofa and placed it in their living room of 20–25 m^2^. They opened the two windows in the living room and the two doors to the adjoining rooms, and these rooms had their windows open. The person used three cans of Guardian textile protection to treat the sofa, but, during application, their airways felt irritated, and the person was affected by the smell. They went outside for fresh air, but coughed continuously for 15–30 min. The person experienced chest pain, got a headache, and was fatigued; therefore, they took paracetamol and ibuprofen. The day after treating the sofa, the person contacted the doctor, and was admitted to hospital. There were no detectable abnormalities on chest X-ray, but the person was still short of breath, especially during physical activity. The person was sent home without treatment, still coughing and with an increased leucocyte count, increased C-reactive protein, and increased lactate dehydrogenase. They had no further decline in health by the next day. The person was a smoker but did not smoke between the application and admission to hospital. 

### 3.2. Aerosol Measurements

**Figure 1:**
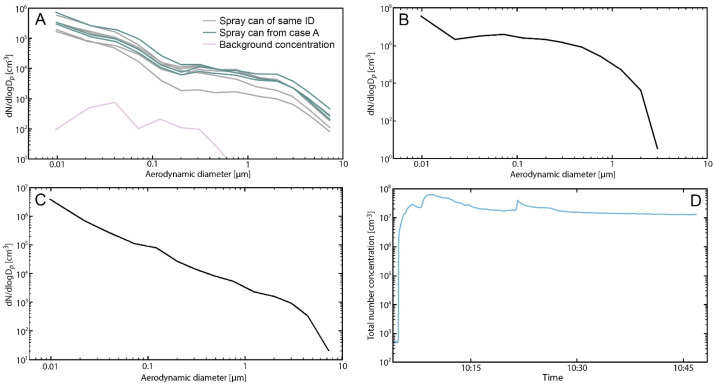
Spray concentrations generated by using the spray can to spray directly into the ELPI inlet (**A**). The green lines are sprays from the can that was used in case A, the black lines are sprays from a can with an identical production number, and purple lines are the background levels in the fume hood. (**B**) Spray concentration measured in the mouse exposure chamber when 0.4 mL/min Guardian textile protection was aerosolised using a pressurised air nebuliser. (**C**) Spray concentrations in a small exposure chamber were generated by spraying with the can. (**D**) Spray concentration in the animal exposure chamber; the initial (10:00 to 10:01) background concentrations in the chamber are shown. After starting the aerosolisation (10:01) of Guardian textile protection at 0.4 mL/min, the particle concentration increased rapidly and was stable throughout the exposure period.

Experiment 1 in the fume hood showed that the spray cans from the same batch produced similar particle number size distributions (Figure 1A). Both cans produced distributions with broad modes of aerodynamic diameters of around <10 nm, 500 nm, and 1 µm. The geometric mean concentration (GM) of the spray cans from the same batch was 1.7 × 10^−8^, and the geometric standard deviation (GSD) was 2.54, for the spray can used in case A, the GM was 1.7 × 10^−8^ and the GSD was 2.63; the background aerosols in the fume hood were insignificant (purple line in Figure 1A). It is clear that the spray events were the source of the particles, as the particle number concentration decreased to background concentrations between each spray event.

Experiment 2 determined the particle number distribution in the mouse exposure chamber (Figure 1B). Prior to exposure onset, the background concentration was <600 particles/cm^3^ (Figure 1D). The generation of aerosols was carried out by infusing Guardian textile protection at a rate of 0.4 mL/min into a pressurised air nebuliser. During the generation of particles, the total number concentrations were >10^7^ particles/cm^3^ (Figure 1D). Particle number concentrations stabilised shortly after the start of exposure and remained at the same level over 45 min; as the particle concentration did not vary significantly, the experiment was stopped (Figure 1D), and the GM was 1.44 × 10^−8^ and the GSD was 2.5. The aerosol size distributions generated using a pressurised air nebuliser were comparable to those generated by using the spray can (Figure 1A and Figure 1B, respectively). The mean mass concentration was 36 mg/m^3^ (assuming spherical particles with a density of 1 g/cm^3^). Almost all of the particles generated were less than 2 µm in diameter and therefore inhalable and able to penetrate deep into the mice’s lungs (Figure 1B). 

The calculated alveolar deposited dose using the average generation rates measured in Experiment 3 (Figure 1C, GM 1.19 × 10^−8^ and GSD 1.7), assuming that the persons sprayed for 30 min, was estimated to be 0.04 mg in both cases. The exposure estimation has been described in detail previously [11]; briefly, the model assumed that the room was 36 m^3^, the air exchange rate was 1.5 h^−1^, the spray time was 30 min, and that the person left the room after spraying. Assuming that none of the alveolar deposited dose was removed during the 30 min exposure, and that there is a total of 1000 mg of lung surfactant in the lungs (for details of this estimation, see [24]), the dose was 0.04 µg/mg lung surfactant.

### 3.3. In Vivo Experiments

**Figure 2:**
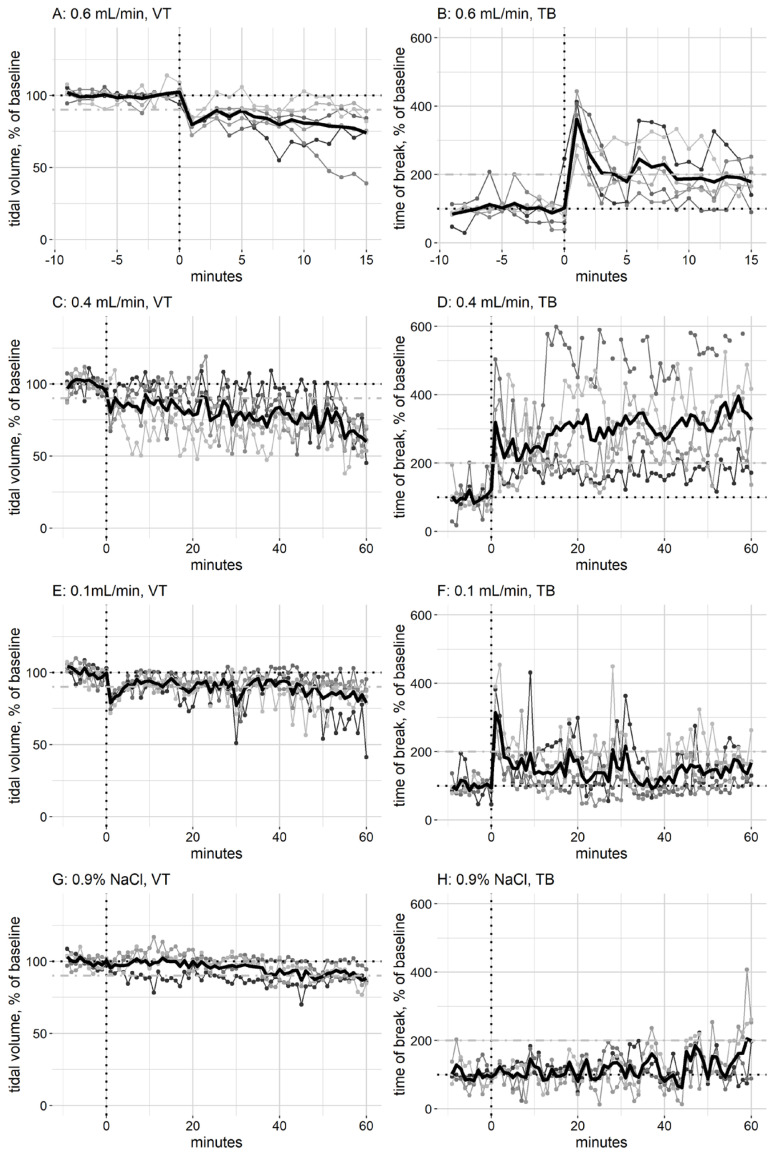
The effect of Guardian textile protection on tidal volume (VT) and time of break (TB) of exposed mice. The mice were allowed to breathe clean air for 10 min (baseline) prior to starting exposure to the product (indicated by a dashed vertical line) (groups of five–six mice); the baseline value is indicated by a dashed black horizontal line. The group average is indicated by a bold black line, and values for individual mice is indicated by a thinner line in grey scale. The effects of sitting in the plethysmograph are illustrated by exposure to 0.9% NaCl (four mice), and the value of the average of the mice exposed to NaCl at the end of 60 min exposure is marked by a dot-dashed grey horizontal line (90% of baseline for VT and 200% of baseline for TB).

The in vivo effect of exposure to Guardian textile protection, as measured by the whole-body plethysmographs, only showed major changes in tidal volume (VT) and time of break (TB), and, therefore, other parameters are not shown. Reduction in VT is the critical effect, as this indicates alveolar collapse. Range finding experiments were performed, with the aim of finding the infusion rate where the respiration was affected. Groups of mice (two–four) were exposed to the product, starting at a very low infusion rate for the first group exposure (0.0003 mL/min), and increasing the infusion rate every 15 min. These experiments were used to determine the infusion rates in the main experiments (0.1, 0.4, and 0.6 mL/min). When mice were exposed to Guardian textile protection at an infusion rate of 0.6 mL/min, there was an immediate drop in VT, and this decline continued throughout the 15 min exposure (Figure 2A). After 15 min of exposure, the VT was significantly different (p 0.032) from the control group exposed to NaCl. The start of exposure also triggered a large increase in TB, and the time between an inhalation and the following exhalation (Figure 2B); however, the TB decreased, and after 15 min of exposure, it was not significantly different from that of mice exposed for 15 min to NaCl (p 0.11). This indicates that the product stimulates the nerves in the upper respiratory tract, triggering sensory irritation [25], but that this irritation reduces after an initial peak. As there was a large effect on VT, the exposure was stopped after 15 min. When mice were exposed to 0.4 mL/min (the lowest observed adverse effect level, LOAEL) of Guardian textile protection, there was a gradual decrease in VT throughout the 60 min exposure (Figure 2C), and after 60 min of exposure, the VT was significantly different (p 0.16) from the control group exposed for 60 min to NaCl. At 0.4 mL/min, there was an immediate large increase in TB that persisted throughout the exposure period (Figure 2D); after 60 min of exposure, the TB was significantly different from the NaCl-exposed mice (p 0.016). However, the effect varied widely between individuals (Figure 2D). At exposure to 0.1 mL/min (no observed adverse effect level, NOAEL), there was an immediate dip in VT at the start of exposure; however, this was recovered shortly after (Figure 2E), and by the end of exposure, the reduction was indistinguishable from the effect of 0.9% NaCl exposure, and there was no significant difference (p 0.905). Similarly, there was an increase in TB at the beginning of the exposure to Guardian textile protection (Figure 2F); however, this also reached baseline levels shortly, and there was no significant difference to exposure to NaCl after 60 min (p 0.730). When mice were exposed to 0.9% NaCl, there was a slight decline in VT and an increase in TB (Figure 2G and Figure 2H, respectively) by the end of the 60 min exposure, indicating that there is an effect on respiration from the strain of being restrained in the plethysmograph, as 0.9% NaCl is not expected to have an effect on respiration. 

Using the respiration data from the in vivo exposure to 0.4 mL/min (LOAEL) (Figure 2C) and combining them with the aerosol concentration in the chamber, assuming a 10% deposition in the alveolar region of the mice and that the mice have 0.15 mg lung surfactant [26], the mice received an alveolar dose of 5.3 µg/mg lung surfactant during the first 5 min of exposure, and a total of 59.7 µg/mg lung surfactant after 60 min exposure. 

### 3.4. In Vitro Experiments

When a cycling drop of lung surfactant was exposed to Guardian textile protection at an infusion rate of 0.4 mL/min, the aerosol inhibited lung surfactant function after 14 ± 8 s of exposure. At the time of inhibition, the deposited dose on the lung surfactant was 27 ± 11 ng/cm^2^, corresponding to 1.08 ± 0.44 µg/mg lung surfactant when using the amount of lung surfactant in each droplet.

## 4. Discussion

This study adds two points to the discussion on pulmonary damage caused by the inhalation of waterproofing spray products such as Guardian textile protection. The in vitro study points to the lung surfactant as a target for the mechanism of action of toxicity [27], and this is further substantiated by the sudden drop in tidal volume when the mice were exposed to the product. When lung surfactant function is inhibited, this leads to collapsed alveoli and a reduction in VT, as described previously in an adverse outcome pathway [14]. Secondly, this study confirms the methods previously used for risk assessment [28]. 

As can be seen from the aerosol experiments, the aerosols produced by the spray can used in case A were identical to the aerosols produced by a can with the same production number (Figure 1A). The spray from the spray product cannot be used to perform experiments where the exposure needs to be controlled and adjustable; thus, the product was aerosolised, after spraying it into a beaker, using a pressurised air nebuliser. This nebuliser produced aerosols of a similar size distribution to the spray can (Figure 1B). However, the nebuliser produced slightly smaller aerosols than those that were produced during spraying using the spray can. Similarly, when the spray can was used in a small exposure chamber to simulate the use of the product, the respirable mass emission rate was 0.04 mg per second and the particle number emission rate was 2.36 × 10^10^ particles per second. This mass emission rate is smaller than similar products tested previously [11]. This is due to the size distribution of the spray, where particles appear mainly as smaller particles with aerodynamic diameters < 0.5 nm. We used the experiment performed in the small exposure chamber (Experiment 3) to estimate the deposited dose in the alveolar region of the two people who were poisoned using the product (case A and B). This assumes that the living room was approximately the same size in case A as in case B. This resulted in an alveolar deposited dose of 0.04 µg/mg lung surfactant for both cases. The mice exposed to 0.4 mL/min Guardian textile protection reacted to the exposure as soon as the exposure started, and the respiratory effects on VT and TB worsened as the exposure continued. During the first 5 min of the animal exposure, the deposited alveolar dose was estimated to be 5.3 µg/mg lung surfactant. This is more than the estimated dose in the human cases; however, the effect in the mice is likely to also be more severe. When the product was tested in vitro, the lung surfactant function was inhibited when 1 µg/mg lung surfactant was deposited on the drop. This suggests that at the exposure both the humans and the experimental animals were exposed to, there was likely an inhibitory effect on the lung surfactant in the intact lungs. A direct comparison between the three different exposures is challenging, and the estimated doses where an effect is seen are surprisingly close to each other. 

The immediate changes in TB in the exposed mice (Figure 2) indicate that the product causes sensory irritation, and this is in line with the two persons complaining of irritation of the airways and pins and needles in the upper airways, signs of respiratory irritation in humans [29].

The label on the product reads “Ensure good ventilation in the room if using indoors. Do not inhale the fumes.” (The full text can be found in Appendix A). The two persons that became ill after using the spray product followed this instruction to their best ability by opening windows and doors. Thus, the two cases suggest that consumer spray products can cause harmful effects, even when the instructions for use are followed. These two cases are not unique, as can be seen from the literature, and this suggests that waterproofing products are difficult to use in a safe way for consumers. 

## 5. Conclusions

We report two cases of poisoning after the use of Guardian textile protection; both persons became ill during or soon after using the product on furniture. The aerosol experiments performed show that aerosols of particle sizes small enough to penetrate deep into the lungs are generated when using the product. These aerosols result in severe changes in the breathing of exposed mice and inhibit lung surfactant function in vitro. This suggests that the mechanism of action at least in part is due to inhibition of lung surfactant function in the lungs. 

## Data Availability

Data are available by contacting corresponding author.

## Appendix A

The label on the Guardian textile protection product reads:

For environmental and safety reasons, this product has been manufactured using Carbon Dioxide as a propellant. Guardian Textile Protection easily and efficiently protects furniture with cloth upholstery and other textiles. Spray approx. 25 cm./10 inches an even layer onto the textile until damp. Allow it to dry for approx. 90 min at minimum 20 °C. If white marks appear on the textile, wipe off with a well-wrung cloth. Ensure good ventilation in the room if using indoors. Do not inhale the fumes. Test always product on a non-visible spot.

Extremely flammable aerosol. Pressurised container: May burst if heated. Protect from sun-light. Do not expose to temperatures exceeding 50 °C/122 °F. Pressurised container: No not pierce or burn, even after use. Keep away from heat, hot surfaces, sparks, open flames and other ignition sources. No smoking. Repeated exposure may cause skin dryness or cracking. If medical advice is needed, have product container or label at hand. Read label before use. Keep out of reach of children. Includes a red diamante with a fire.
